# Relationship between ER expression by IHC or mRNA with Ki67 response to aromatase inhibition: a POETIC study

**DOI:** 10.1186/s13058-022-01556-6

**Published:** 2022-09-12

**Authors:** Elena Lopez-Knowles, Simone Detre, Margaret Hills, Eugene F. Schuster, Maggie C. U. Cheang, Holly Tovey, Lucy S. Kilburn, Judith M. Bliss, John Robertson, Elizabeth Mallon, Anthony Skene, Abigail Evans, Ian Smith, Mitch Dowsett

**Affiliations:** 1 The Breast Cancer Now Toby Robins Research Centre, The Institute of Cancer Research, London, UK; 2 Ralph Lauren Centre for Breast Cancer Research, Royal Marsden Hospital, London, UK; 3 Clinical Trials and Statistics Unit, Division of Clinical Studies, The Institute of Cancer Research, London, UK; 4 Graduate Entry Medical School, Royal Derby Hospital, University of Nottingham, Uttoxeter Road, Derby, DE22 3DT UK; 5 Queen Elizabeth University Hospital Glasgow, Govan, UK; 6 University Hospitals Dorset (Royal Bournemouth), Bournemouth, UK; 7 University Hospitals Dorset (Poole), Poole, UK; 8 Royal Marsden Hospital, London, UK

**Keywords:** Breast cancer, ESR1, PgR, Aromatase inhibitors, Ki67

## Abstract

**Background:**

In clinical practice, oestrogen receptor (ER) analysis is almost entirely by immunohistochemistry (IHC). ASCO/CAP recommends cut-offs of < 1% (negative) and 1–10% (low) cells positive. There is uncertainty whether patients with ER low tumours benefit from endocrine therapy. We aimed to assess IHC and mRNA cut-points for ER versus biological response of primary breast cancer to 2 weeks’ aromatase inhibitor treatment as measured by change in Ki67.

**Methods:**

Cases were selected from the aromatase inhibitor treatment group of POETIC. We selected the 15% with the poorest Ki67 response (PR, < 40% Ki67 suppression, *n* = 230) and a random 30% of the remainder categorised as intermediate (IR, 40–79% Ki67 suppression, *n* = 150) and good-responders (GR, ≥ 80% Ki67 suppression, *n* = 230) from HER2 − group. All HER2 + cases available were selected irrespective of their response category (*n* = 317). ER expression was measured by IHC and qPCR.

**Results:**

ER IHC was available from 515 HER2 − and 186 HER2 + tumours and ER qPCR from 367 HER2 − and 171 HER2 + tumours. Ninety-one percentage of patients with ER IHC < 10% were PRs with similar rates in HER2 − and HER2 + cases. At or above ER IHC 10% substantial numbers of patients showed IR or GR. Similar proportions of patients were defined by cut-points of ER IHC < 10% and ER mRNA < 5 units. In addition, loss of PgR expression altered ER anti-proliferation response with 92% of PgR − cases with ER IHC < 40% being PRs.

**Conclusions:**

There was little responsiveness at IHC < 10% and no distinction between < 1% and 1–10% cells positive. Similar separation of PRs from IR/GRs was achieved by IHC and mRNA.

**Supplementary Information:**

The online version contains supplementary material available at 10.1186/s13058-022-01556-6.

## Background

Annually over 2 million women are estimated to develop breast cancer worldwide [1]. The presence or absence of significant expression of oestrogen receptor alpha (ER) is the key determinant of whether these patients should receive endocrine therapy [2]. Methodologies for measuring the degree of expression of ER have changed markedly since it became widely measured in the late 1970s and early 1980s by one of a number of ligand-binding assays (LBAs) most often by the dextran-coated charcoal assay (DCC) [3]. At the end of the 1980s, antibodies began to be used to measure ER in quantitative enzyme-linked immunoassays (ELISAs) [4]. These were soon superseded by immunohistochemical assays (IHCs). Current measurement for clinical diagnostics is almost universally by IHC.

Meta-analysis of numerous randomised clinical trials of adjuvant tamoxifen versus no tamoxifen conclusively revealed that patients with tumours with < 10fmol ER/mg protein when measured by LBA/DCC gained no significant reduction in risk of recurrence from tamoxifen treatment [5]. In contrast, the subgroup with 10–19 fmol/mg protein showed a 33% reduction in risk. Cut-points for IHC have been developed largely by calibrating IHC expression against values from LBAs leaning towards lower cut-points to minimise the exclusion of patients that might gain some benefit from endocrine therapy. This approach underpinned the most recent ASCO/CAP guidelines, which recommended that tumours with < 1% of cells staining positive should be considered ER-negative [6].

For the first time, the guidelines formally established that the group with 1–10% staining should be considered a distinct category described as “ER Low”. The limited data on benefit from endocrine therapies that underpin that designation significantly impairs the confidence with which oncologists manage patients with such tumours.

Some studies suggest that mRNA levels if calibrated against benefit from endocrine treatment could prove a preferable means of analysis [7], but such measurement is not recommended by ASCO/CAP for diagnostics. Assessment of mRNA levels requires no more material than IHC, and it provides quantitative results. Methodologies have been developed that could enable the measurement of mRNA levels without requiring the expert analysts and equipment needed for IHC [8]. One small study concluded that data on ESR1 expression by qPCR showed a continuous relationship with decreased risk of recurrence with tamoxifen that was not apparent with data from IHC [9]. Unfortunately, that study excluded cases in which the tumour was < 10fmol/mg protein and was too small for generalisability. There seems little prospect of studies of archival material from randomised trials of tamoxifen versus no tamoxifen to enable studies of sufficient size to provide clinical confidence. Developing a cut-point for mRNA from the many studies of correlations with IHC data, which are themselves not directly related to clinical outcome, seems unadvisable.

The change in Ki67 levels as a result of short-term treatment with endocrine treatment is an attractive end-point for assessing the biological responsiveness of primary tumours to a given therapy and has been shown to be related to the reduction in risk of recurrence from those treatments [10]. We have therefore aimed to (i) describe the relationship of cut-points for ER with IHC or qPCR with Ki67 response to short-term treatment of primary breast cancers with a non-steroidal aromatase inhibitor, (ii) determine whether cut-points for IHC or qPCR were related to Ki67 response more closely than the other or whether the addition of qPCR to IHC could improve that relationship and (iii) to determine whether these relationships differed according to HER2 and PgR status of the tumours.

## Methods

### Patient characteristics

Formalin-fixed, paraffin-embedded (FFPE) tumour samples were collected as part of the PeriOperative Endocrine Therapy-Individualising Care (POETIC) trial (CRUK/07/15) from postmenopausal women with primary ER + breast cancer. For eligibility to the trial, ER status was determined locally. Full details of the eligibility criteria, the conduct of the trial and its primary outcome data are published elsewhere [11]. In brief, the trial randomised 4,480 postmenopausal women recruited between 2008 and 2014 to receive aromatase inhibitor (AI) 2 weeks before and 2 weeks after surgery or no perisurgical treatment. Patients were recruited irrespective of HER2 status, which was also established locally by immunohistochemistry and fluorescent in situ hybridisation on the surgery sample. Tumours needed to be palpable or at least 1.5 cm by ultrasound.

In this exploratory substudy, only baseline samples with Ki67 ≥ 10% (to maximise the precision of estimates of AI-induced proportional changes) were selected from the AI-treatment group. These were categorised by (i) HER2 status and then (ii) by degree of Ki67 response as a percentage of baseline after 2 weeks of AI. For HER2 − cases, all samples from the 15% worst responders (PR), 30% of the 35% of patients with intermediate response (IR) and 30% of the 50% best responders (GR) were analysed. Samples for all 3 groups in this HER2 − subset were matched by baseline Ki67 within groups 10–20%, 20–30%, 30–40% or 40 + %. All HER2 + samples were analysed, and the same Ki67 cut-offs were applied to define PR, IR and GRs.

Tissue for IHC and mRNA were taken as sequential sections from the same block.

### Immunohistochemical methodology

Staining was undertaken at the Ralph Lauren Centre for Breast Cancer Research. ER and PgR staining were analysed in the baseline sample, whilst Ki67 staining was evaluated in the baseline and 2-week sample. ER staining was done using the ER antibody (NCL-L-ER6F11, Novocastra, Leica) at 1:400 dilution (8.5 µg/ml) and PgR antibody (L-PgR-312 clone 16, Novocastra, Leica) at 1:300 dilution (11.6 µg/ml). Antigen retrieval for both was done in Envision FLEX Target Retrieval Solution low PH (DM829, Dako Agilent) in a Dako PT Link Tissue Processor at 97 °C for 20 min. The Envision FLEX detection system on the Autostainer from DAKO was used. Ki67 staining was done with the Anti-MIB1 clone (M7240, Agilent DAKO UK).

The ER scoring method was adapted from a global scoring protocol that has been developed by the IKBCWG for Ki67 scoring [12, 13]. The whole section was examined in order to estimate the percentages of the invasive tumour component exhibiting relatively high, medium, low or negligible ER staining frequencies. Based on these estimates, the assessor decided on which fields to score for each ER staining distribution in the whole tumour. In total, four representative high-power fields (HPF) of invasive breast cancer were selected and in each HPF, 100 invasive tumour cells were scored. The number of ER-positive nuclei was counted irrespective of the staining intensity. ER positivity was calculated as the percentage of the total number of ER-positive invasive tumour cells in all assessed fields relative to the total number of invasive tumour cells. Where there were no positive cells or the score was < 1%, the presence or absence of normal/benign ducts was noted and whether they contained ER-positive nuclei; if tissue was available the staining/scoring of cases scored as < 1% was redone to confirm the negative result and to avoid the possibility that this was a false-negative/low result in the central laboratory.

The percentage of PgR positivity was assessed by visual estimation using a light microscope. The whole section was examined at low, medium and high power in order to estimate manually by eye the % PgR-positive nuclei present in the invasive breast cancer. PgR was deemed positive when ≥ 1% of cells were positive.

### RNA extraction and cDNA synthesis

RNA was coextracted with DNA from three 10 µm FFPE sections from the baseline block of patients using the ROCHE High Pure miRNA isolation kit for RNA (Roche, Basel, Switzerland) and the Allprep FFPE kit for DNA (Qiagen) following SOP M027 from The Cancer Genome Atlas (TCGA) Program developed by the Biospecimen Core Resource (BCR) at Nationwide Children’s Hospital in Columbus, Ohio. Quantitation was done using the high-sensitivity RNA Qubit assay (Thermo Fisher Scientific, Carlsbad, CA).

Two hundred nanograms of RNA was reverse-transcribed using SuperScript IV VILO Master mix (Thermo Fisher Scientific, Carlsbad, CA) following the manufacturer’s instructions, with thermocycling conditions: 25 °C for 10 min, 50 °C for 10 min and 85 °C for 5 min.

### Quantitative PCR

*ESR1*, *ACTB* and *TFRC* levels were measured by RT-qPCR based on TaqMan commercial probes in a QuantStudio 6 FLEX PCR Detection System (Applied Biosystems, Foster City, CA). Hs00951083_m1 (*TFRC*), Hs01060665_g1 (*ACTB*) and Hs01046816_m1 (*ESR1*) were the probes used. Data analysis was performed with QuanStudio FLEX software V.1.7.1.

The RT-qPCR mix was prepared according to TaqMan Fast Universal PCR Master Mix instructions (Applied Biosystems, Foster City, CA). The PCR mix contained 2.5 µl Universal Master Mix, 0.25 µl of the Assay of interest, 0.25 µl of Water and 10 ng of cDNA. The reactions were performed in triplicate, and any of these with standard deviation > 0.3 was excluded. The PCR cycler conditions were the same for all 3 assays: 50 °C 2 min, 95 °C for 5 min and 40 cycles of 95 °C for 2 s and 60 °C for 25 s.

The relative mRNA level of *ESR1* was determined as 2^^−Delta Ct^ (2^-(Ct *ESR1* in test – Ct average of *ACTB*/*TFRC* in test)). The primary result of a real-time PCR is a ct value, and by calculating the delta-ct value we obtain the relative mRNA level measured as units of relative expression.

### Statistical analyses

Ki67 residual percentage is calculated by dividing Ki67 expression levels in the surgery sample by Ki67 expression levels in the baseline sample. Spearman rank correlation was used to analyse the correlations between ER, PgR, Ki67 residual percentage and *ESR1* mRNA levels. This analysis results in a rho value, which measures the strength of the association between the two variables analysed. For IHC, comparisons were made between ER categories of < 1%, ≥ 1 < 10 and ≥ 10%. Among the cases ≥ 10%, further categories were considered as follows: ≥ 10 < 20, ≥ 20 < 40, ≥ 40 < 60, ≥ 60 < 80 and ≥ 80.

As described above, ALL 15% poor responder patients from the HER2 − POETIC patient group were selected; however, only 30% of the 35% intermediate and 50% good responders were selected due to the time/cost constraints of analysing over 2000 patients if we had analysed the whole HER2 − POETIC population. Thus, when making comparisons between the prevalence of PR vs GR and/or IR, the number of cases that were GR or IR was multiplied by 3.3 to account for our assessing only 30% of that category. This results in some of the estimated total cases being non-integer. Cut-points would be identified according to the proportion of PRs, with at least 90% being considered desirable.

## Results

### Patient population

A consort diagram of available samples is shown in Additional file 1: Figure S1. From the 2607 HER2 − POETIC cases, those with at least 10% Ki67 at baseline were selected according to Ki67 response: the 15% worst responders, 30% of the 50% best responders and 30% of the 35% intermediate responders. From the 317 HER2 + POETIC cases, all cases with at least 10% Ki67 at baseline were selected irrespective of their response group. FFPE baseline blocks from 722 tumours (534 HER2 − and 188 HER2 +) with Ki67 values at both baseline and surgery were selected for analysis. Results were obtained from 701 tumours for IHC and 538 for mRNA. Table 1 describes the clinicopathological information of the overall and subpopulations. Overall, the population had a median age of 67 and were mainly small, ductal, grade 2 tumours, with no vascular invasion.

**Table 1:** Clinicopathological characteristics of the overall population and the HER2 − and HER2 + subsets

	HER2 − (*n* = 515)	HER2 + (*n* = 186)	TOTAL (*n* = 701)
*Age (years)*			
Median	68	66	67
< 59	102 (19%)	33 (18%)	135 (19%)
60–69	183 (35%)	86 (46%)	269 (38%)
70–79	158 (30%)	52 (28%)	210 (31%)
≥ 80	72 (14%)	15 (8%)	87 (12%)
*Tumour size (cm)*			
Median	2	2	2
≤ 2	333 (64%)	124 (67%)	457 (65%)
> 2 & ≤ 5	175 (34%)	57 (31%)	232 (33%)
> 5	5 (1%)	3 (2%)	8 (1%)
Unknown	2 (1%)	2 (1%)	4 (1%)
*Tumour grade*			
G1	40 (8%)	4 (2%)	44 (7%)
G2	282 (55%)	85 (46%)	367 (53%)
G3	146 (28%)	80 (43%)	226 (31%)
Unknown	47 (9%)	17 (9%)	64 (9%)
*Nodal status*			
0	296 (57%)	104 (56%)	400 (57%)
≥ 1	219 (43%)	82 (44%)	301 (43%)
*Vascular invasion*			
No	314 (61%)	106 (57%)	420 (60%)
Yes	175 (34%)	74 (40%)	249 (35%)
Unknown	26 (5%)	6 (3%)	32 (5%)
*Histological type*			
IDC	441 (86%)	164 (88%)	605 (86%)
ILC	57 (11%)	14 (7%)	71 (10%)
Other	12 (2%)	5 (3%)	17 (3%)
Unknown	5 (1%)	3 (2%)	8 (1%)
*PgR % baseline*			
Median	50	20	40
< 1%	94 (18%)	63 (34%)	157 (22%)
≥ 1%	421 (82%)	123 (66%)	544 (88%)
*Ki67% baseline*			
Median	26	30	27
*Ki67 response categories*			
GR	198 (38%)	47 (25%)	245 (35%)
IR	124 (24%)	69 (37%)	193 (28%)
PR	193 (38%)	70 (38%)	263 (37%)

All PR tumours had Ki67 suppression of < 40% with a median 6% decrease (IQR -16.6% to + 26%). IR tumours had suppression of 40 to 79% with a median of 65% (IQR 53% to 74%). GR tumours had at least 80% suppression and a median of 90% (IQR 85% to 95%).

### Immunohistochemistry

Seven hundred and one tumours were scored for ER. Tumours were classified into three subgroups based on ER IHC levels: < 1% (negative), ≥ 1 to 10% (low) and ≥ 10% (positive). The clinicopathological characteristics of tumours in these separate categories are shown in Additional file 1: Table S1.

Seven hundred and one tumours were also scored for PgR. Median score for the overall population was 40% (IQR 1% to 85%). HER2 − population had a median PgR score of 50% (IQR 5% to 90%), and the HER2 + population had a median score of 20% (IQR 0% to 69%).

### ER IHC and response to AI treatment

**Fig. 1:**
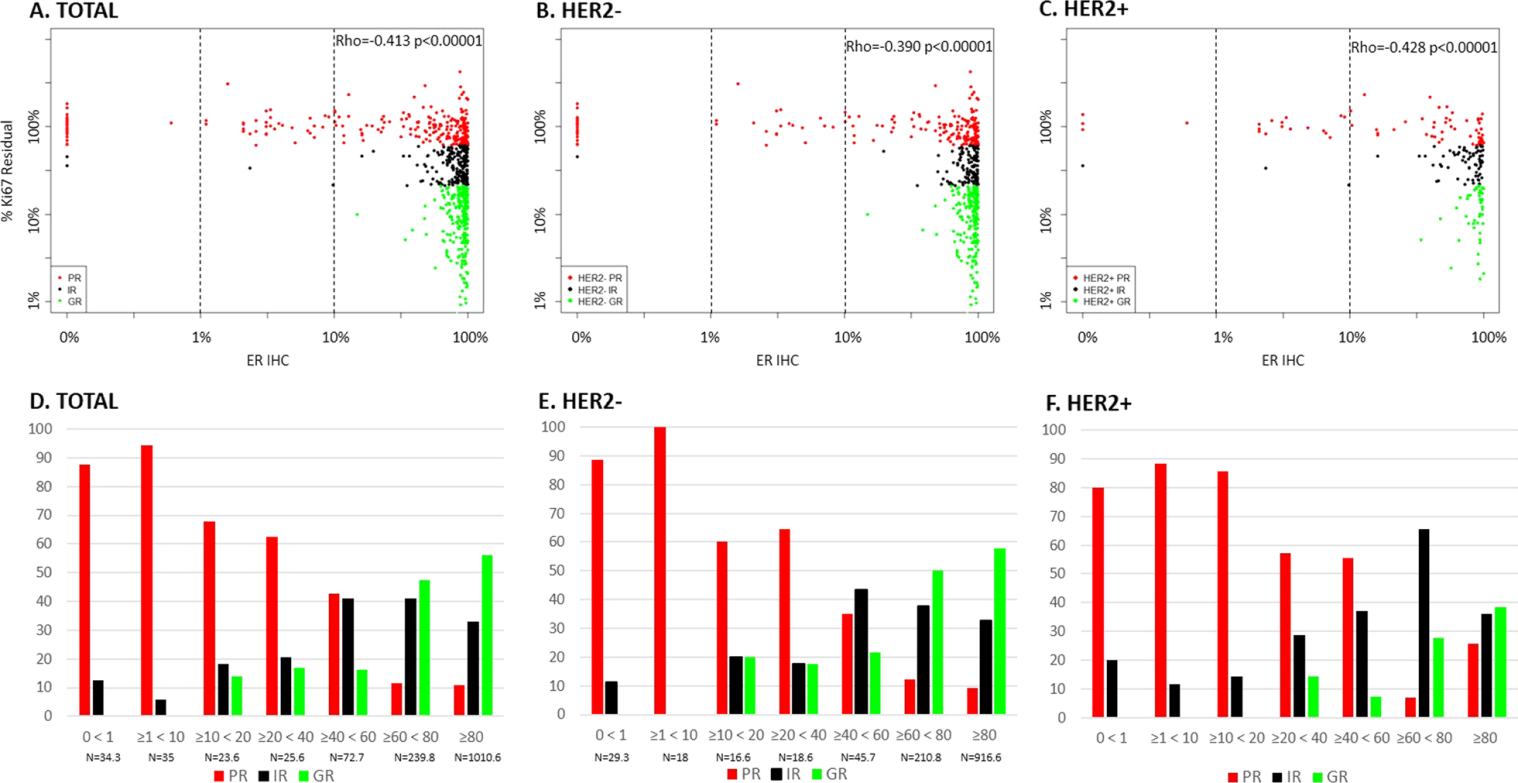
ER IHC in relation to Ki67 residual % (Ki67 at surgery*100/baseline) by HER2 status. Scatterplots of ER IHC measured in baseline tissue in relation to Ki67 in the overall population (**A**), HER2 − (**B**) and HER2 + (**C**) (original numbers, *n* = 701) and percentage of patients in Ki67 response categories (PR, IR, GR) in the overall population (**D**), HER2 − (**E**) and HER2 + (**F**) distributed into ER IHC subgroups (HER2 − GR and IR are multiplied by 3.3; derived numbers, *n* = 1441.6). Red is PR, Black is IR and Green is GR

The relationship between ER by IHC and Ki67 response to AI measured after 2 weeks of treatment is shown in Fig. 1 for the overall, HER2 − and HER2 + populations. The Rho values of the overall population and the HER2 − and HER2 + populations were relatively similar (Rho = − 0.413, Rho = − 0.390 and Rho = − 0.428, respectively). None of the ER-negative or ER-low cases were GRs, and there were just 2 IRs in each of those ER categories. After adjusting for the selection of 30% of the IRs and GRs, 30.0/34.3 (87%) ER negative and 33.0/35.0 (94%) ER low cases were PRs (Fig. 1 and Additional file 1: Table S2). Thus, there was little evidence of AI responsiveness for ER < 10%. The cut-point of < 10% vs ≥ 10% defined 4.8% of the population as below the cut-point. The percentage of patients showing PR decreased to 68% (16.0/23.6) for cases with ER ≥ 10%, < 20% and 62.5% (16.0/25.6) for cases with ER ≥ 20%, < 40%. Thus, there was substantial evidence of responsiveness in these ER IHC categories although only 15% (7.6/49.2) were GR for ER ≥ 10%, < 40%. The degree of responsiveness increased with increasing ER such that for the ER ≥ 80% category 56% (567.3/1010.6) of cases were GR. The relationship between ER by IHC and Ki67 responsiveness differed with a lower proportion of GRs in the higher ER categories.

For HER2 + cases, the proportion of PRs and IRs did not differ until ER levels were at least 20%, but given the small number of HER2 + cases in the individual lower ER categories there was no clear evidence for different cut-points for HER2 + and HER2 − tumours (Fig. 1B and C).

**Fig. 2:**
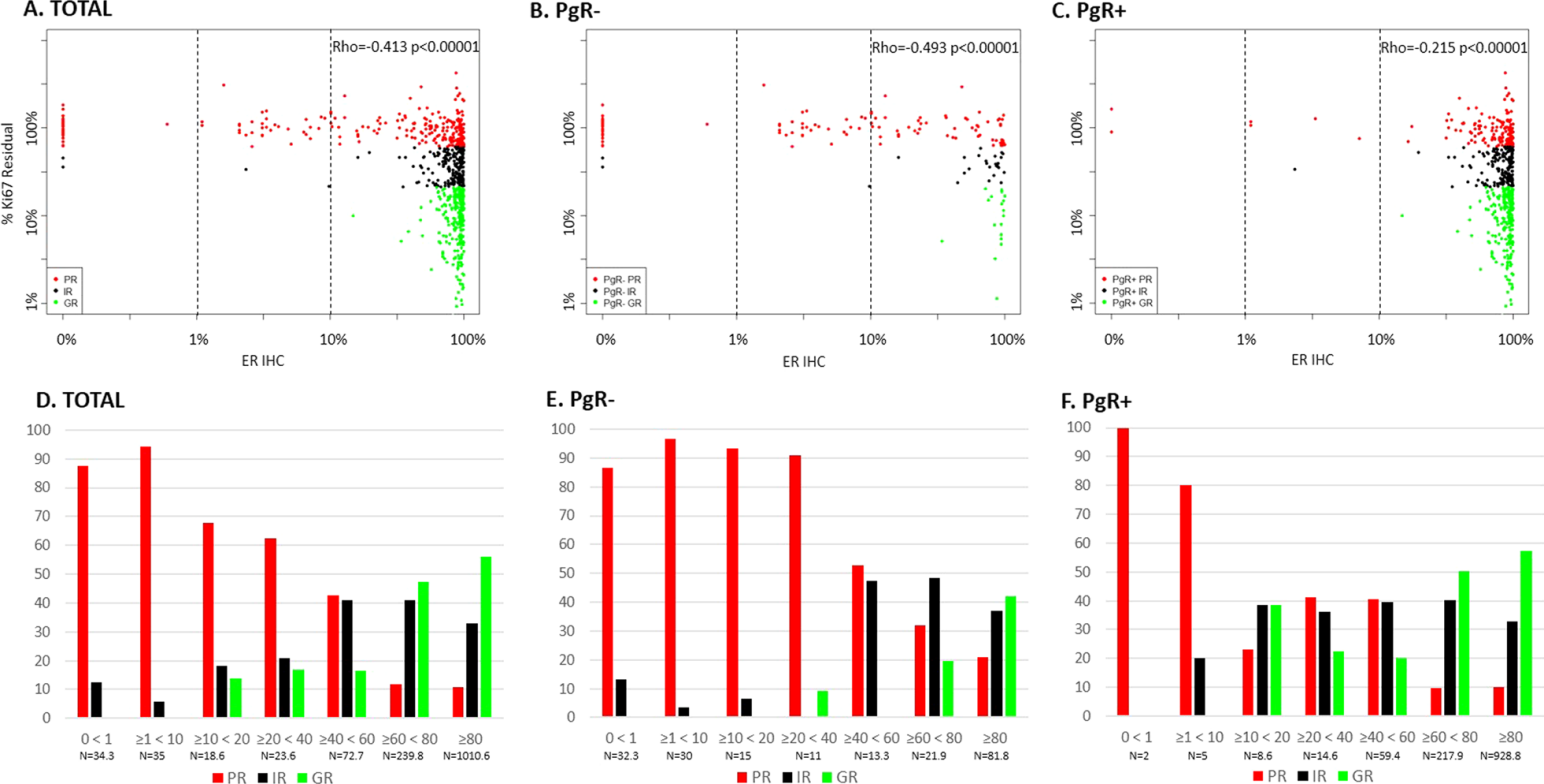
ER IHC in relation to Ki67 residual % (Ki67 at surgery*100/baseline) by PgR status. Scatterplots of ER IHC measured in baseline tissue in relation to Ki67 in the overall population (**A**), PgR − (**B**) and PgR + (**C**) (original numbers, *n* = 701) and percentage of patients in Ki67 response categories (PR, IR, GR) in the overall population (**D**), PgR − (**E**) and PgR + (**F**) distributed into ER IHC subgroups (HER2 − GR and IR are multiplied by 3.3; derived numbers, *n* = 1441.6). Red is PR, Black is IR and Green is GR

The relationship between ER by IHC and Ki67 response is shown according to PgR (IHC) in Fig. 2A–C. As expected, very few of the ER-negative or ER-low patients were PgR + (Fig. 2C). The prevalence of PRs was higher across all ER categories for the PgR − than the PgR + cases. Even between ER levels between 10 and 40% positive, the frequency of IR or GR combined was just 8% (2/26) for PgR − cases compared with 66% (15.2/23.2) for PgR + (Additional file 1: Table S3). For PgR − tumours with < 40% ER, 92% (81/88.3) of cases were PRs. Thus, the threshold for responsiveness was higher in the PgR − cohort at approximately 40% rather than 10% in the PgR + .

If PgR positivity was defined as ≥ 10% rather than ≥ 1% the distribution of PR, IR and GR, the relationship between ER and Ki67 response was largely similar (Additional file 1: Figure S2).

### ER mRNA and response to AI treatment

**Fig. 3:**
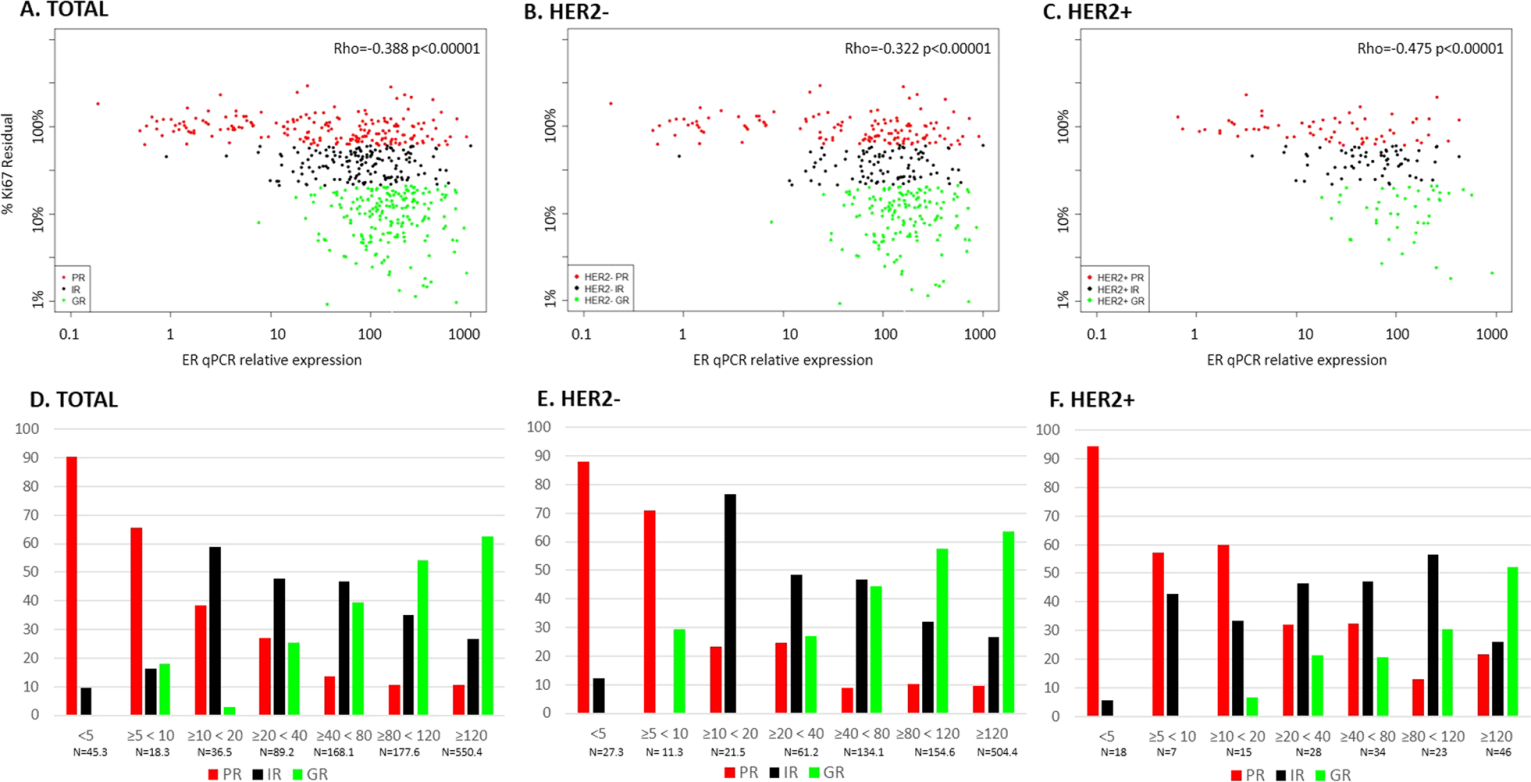
ER qPCR in relation to Ki67 residual % (Ki67 at surgery*100/baseline) by HER2 status. Scatterplots of ER qPCR measured in baseline tissue in relation to Ki67 in the overall population (**A**), HER2 − (**B**) and HER2 + (**C**) (original numbers, *n* = 538) and percentage of patients in Ki67 response categories (PR, IR, GR) in the overall population (**D**), HER2 − (**E**) and HER2 + (**F**) distributed into ER qPCR subgroups (HER2 − GR and IR are multiplied by 3.3; derived numbers, *n* = 1085.4). Red is PR, black is IR and green is GR

The relationship between ER by mRNA and Ki67 response to AI measured after 2 weeks of treatment is shown in Fig. 3 for the overall, HER2 − and HER2 + populations. The Rho values of the overall, HER2 − and HER2 + populations were − 0.388, − 0.322 and − 0.475, respectively.

Overall, 91% patients (41/45.3) with ER mRNA relative expression < 5 were PR and none were GR, irrespective of their HER2 status (Additional file 1: Table S4). At a relative expression cut-point of < 5 vs ≥ 5, 4.2% (45.3/1085.4) of the analysed population fell below the cut-point. The PR percentage was 65% (12.0/18.3) overall for the ER group ≥ 5, < 10 and 38% (14.0/36.5) for the ER group ≥ 10, < 20. 5.9% (63.6/1085.4) of the population fell below the relative expression cut-point of < 10 vs ≥ 10, with 11.5% being IRs and 5.2% being GRs.

Similar to the IHC data, the distribution of PR, IR and GRs was relatively similar between the HER2 − and HER2 + cases, but there were fewer GRs in almost all of the ER categories for the HER2 + population.

**Fig. 4:**
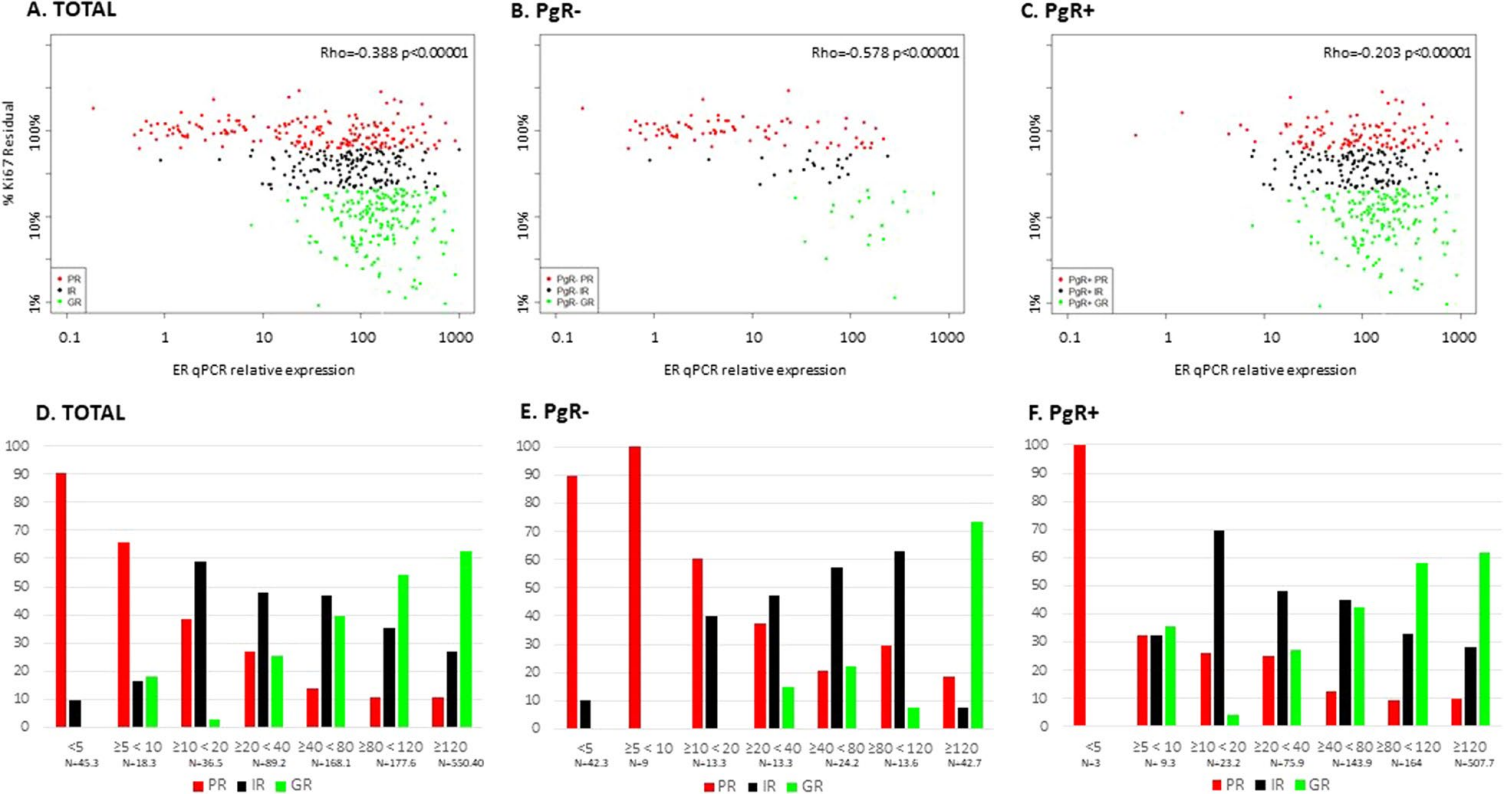
ER qPCR in relation to Ki67 residual % (Ki67 at surgery*100/baseline) by PgR status. Scatterplots of ER qPCR measured in baseline tissue in relation to Ki67 in the overall population (**A**), PgR − (**B**) and PgR + (**C**) (original numbers, *n* = 538) and percentage of patients in Ki67 response categories (PR, IR, GR) in the overall population (**D**), PgR − (**E**) and PgR + (**F**) distributed into ER qPCR subgroups (HER2 − GR and IR are multiplied by 3.3; derived numbers, *n* = 1085.4). Red is PR, black is IR and green is GR

Among PgR − tumours, there were no GRs with relative ER qPCR expression < 20 and among PgR + tumours GRs occurred with relative ER qPCR as low as 5–10 (Fig. 4, Additional file 1: Table S5). While these data suggest that cut-points for relative expression higher and lower than 10 might be appropriate for PgR − and PgR + tumours, respectively, the number of cases available to address this within the subgroups was too small to provide confidence.

When PgR positivity is defined as ≥ 10% rather than ≥ 1%, the distribution of PR, IR and GR shows a similar relationship between ER and Ki67 response (Additional file 1: Figure S3).

### Immunohistochemistry and mRNA correlation

**Fig. 5:**
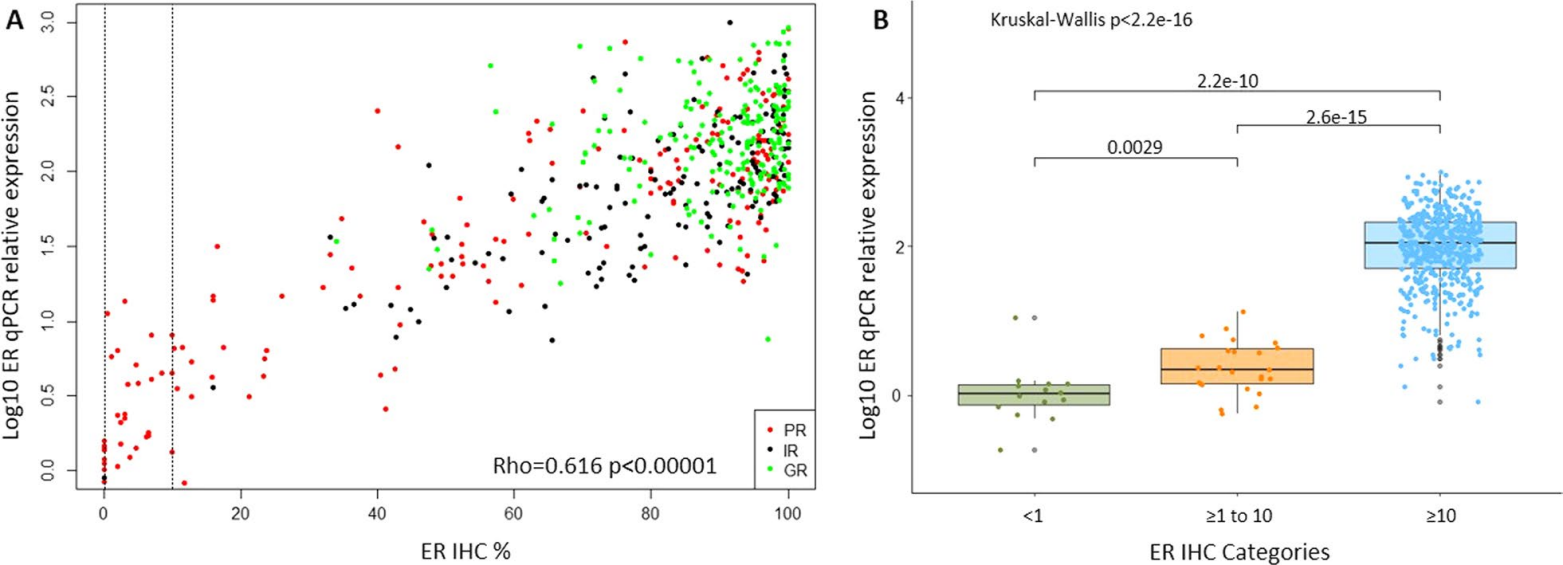
ER IHC and qPCR. **A** Scatterplot of Log10 ER qPCR in relation to ER IHC percentage measured in baseline tissue (original numbers, *n* = 538). **B** Boxplot of Log10 ER qPCR divided into 3 ER IHC categories: < 1%, ≥ 1 to 10% and ≥ 10% (original numbers *n* = 538)

The correlation between ER levels by IHC and qPCR is shown in Fig. 5A (Rho = 0.611, *p* < 0.00001). ER mRNA was detectable for cases in the < 1% IHC category (Fig. 5B). Levels of mRNA were significantly lower than in the ≥ 1%, < 10% IHC group. There was a small overlap in mRNA levels between the latter and those in the ≥ 10% IHC, but the overall difference was nearly 2 orders of magnitude.

We wished to address the question of whether the conduct of qPCR could distinguish PRs from IRs or GRs among the cases that were ER low by IHC. However, in the dataset with both qPCR and IHC results, all ER low cases were PRs. In the tumours with ER IHC between 10 and 40%, there was some indication that those with higher qPCR levels had a greater chance of being IR or GR, but the number of cases was too low to create any cut-off rules with confidence.

## Discussion

Given the clinical effectiveness and general good tolerability of endocrine therapy, identifying those patients with primary breast cancer that have minimal chance of benefit has been an important goal for many years. As explained above, while there is a compelling amount of evidence for a cut-point of 10 fmol/mg protein with the now unused LBA (EBCTCG) [5], there is little direct evidence for the 1% and 10% cut-points that underpin IHC for ER. We aimed to determine the biological responsiveness of samples with low-to-moderate levels of ER by IHC and mRNA to AIs using short-term change in Ki67 as the end-point, given that this change had been shown to reflect the degree of benefit from endocrine treatment in clinical trials (IMPACT, POETIC).

Using central IHC testing in our set of patients from the treated arm of the POETIC trial, 5% were ER negative (< 1%), 5% ER low (≥ 1 to 10%) and 90% ≥ 10% ER high. The very low proportion of ER negatives was largely due to the POETIC trial being limited to ER + tumours as measured at local centres. To ensure that we captured as many patients with low-to-moderate levels of ER, we selected all patients for study with the 15% of poorest Ki67 response. This group had a median suppression of only 6% relative to baseline. We analysed 30% of the rest of the HER2 − population given the expectation of much higher levels of ER, and this was borne out in our study. All HER2 + tumours were included since they make up only about 10% of the ER + population. Despite this approach to enriching for lower values, the proportion of cases that have < 50% positive cells is modest due to the widely recognised largely dichotomous distribution of ER by modern IHC [14, 15]. We corrected for these differences in samples analysed when assessing the prevalence of PR, IR and GR in different cohorts of ER positivity.

Our data indicated that there is very little relevant biologic responsiveness for tumours with < 10% cells staining but substantial increasing responsiveness with increasing levels of ER at or above 10%. A cut-off of 10% segregated a cohort in which 91% were PRs; this is consistent with the philosophy of minimising the proportion of responsive patients below the cut-off. Two weeks might be considered a relatively short time to achieve a significant reduction in Ki67. However, while Ki67 suppression does increase in some patients between 2 and 12 weeks on AI, there are very few patients that show sufficient change to convert them from PR into a GR [10].

A 10% cut-off was equally applicable for HER2 − and HER2 + tumours with HER2 positivity appearing to impede responsiveness across the range of ER. In contrast, the data indicated that very little responsiveness below a cut-point of 40% for PgR − tumours, irrespective of the use of a 1% or 10% cut-point for PgR positivity. Almost all patients with HER2 + disease receive adjuvant anti-HER2 therapy, and most receive adjuvant chemotherapy; adjuvant endocrine therapy is given after the chemotherapy is complete. The impact of endocrine therapy would therefore be on any residual micrometastatic disease. Currently, there are no means to access those micrometastases for biomarker analyses of the type made here.

There is substantial interest in the potential use of mRNA to supplant IHC for clinical diagnostics. mRNA is generally a more robust and reliable measure of quantification and has been shown to be highly correlated with ER IHC in a number of studies [16, 17]. Consideration has been given to whether mRNA might provide a more accurate assessment of ER expression in ER low tumours. Singh et al. reported [16] that all ER + by IHC were ER + by qPCR; however, 56% of ER low by IHC were negative by qPCR. This confirmed a previous study where low ER tumours tended to be ER negative by ESR1 mRNA. While the tumours were predominantly luminal, they showed low predicted endocrine sensitivity by the SET gene signature [18].

Our data indicated that mRNA levels were correlated with Ki67 response across the whole range of ER levels as well as IHC analyses. There was little evidence that follow-up mRNA analyses could improve the segregation of responsive and non-responsive patients in those with ER by IHC < 10%. Units for mRNA measurement by qPCR are essentially arbitrary and to create a meaningful cut-off that could be used between centres and/or methodologies would require the availability of a well-validated material for calibration. Using the scale that we chose, PRs made up 91% of the cases with < 5 units, and this identified 4.2% of the population with such poor responsiveness compared with 4.8% by IHC.

Both the mRNA and IHC data could be used to identify patients with different levels of resistance/responsiveness than the 90%/10% values used here, for example in clinical trials using presurgical exposure to endocrine agents to select appropriate patients for additional therapy [19, 20]. Notably, when selecting patients that could be deferred from surgery during the COVID pandemic, a cut-point of 40% for ER was identified but that was in association with the 2-week value of Ki67 rather than its proportional suppression.

Others have taken different approaches to characterise the ER group with 1–10% cells positive. The largest recent study described that overall, the clinicopathological characteristics of the ER low showed they were distinct from the ER-positive cancers but shared features with the ER-negative cancers. They were associated with higher grade, negative PgR and positive HER2 status. Prognostically, however, ER-positive low were similar to ER-positive tumours [21]. Another recent study focusing on HR low (ER and PgR) showed that patients with HER2 − HR-low tumours had significantly worse survival than HER2 − HR-positive tumours; by contrast in HER2 + tumours no difference was observed between the 3 HR groups [22]. A further study showed low ER patients do not respond well to endocrine therapies and have a pathologic complete response similar to TNBC in the neoadjuvant setting [23]. Smaller studies have found essentially similar results [24–26]. A deficit of each of these studies is the absence of a measure of the responsiveness of the tumours.

The strengths of our study include all analyses being conducted by central analysis, the relatively large size of the population studied, the derivation of the samples from a well-conducted randomised trial and the enrichment of cases with low-to-moderate ER levels, given the low prevalence of these. The main limitations are that only patients with ER + tumours according to local practice were included and that change in Ki67 is an index of clinical response rather than clinical response per se, albeit that the two are strongly correlated. Although this study drew its cases from a large clinical trial, the relatively low proportion of patients with ER-low tumours in this study, which is similar to that seen in routine practice, did not allow for a test and validation type approach to create an optimum cut-point. Rather for the IHC study, we used the 1% and 10% cut-points that were defined by ASCO/CAP as their negative/low and low/high cut-points, respectively. We did not undertake an assessment of ER levels versus clinical outcome in POETIC since this could reflect the relationship with prognosis not response to endocrine treatment.

## Conclusions

In summary, ER IHC levels below 10% have very limited antiproliferative response to AIs in both HER2 − and HER2 + disease. For PgR − tumours, little response is seen below 40%. ER mRNA analyses can identify a very similar size cohort of poorly responsive patients.

## Supplementary Information

**Additional file 1: **Supplementary Figures and Tables.

## Data Availability

Data were generated by the authors and available on request.

## Abbreviations

BC: Breast cancer
ER: Oestrogen receptor
HER2: Human epidermal growth factor receptor 2
IHC: Immunohistochemistry
mRNA: Messenger RNA
PgR: Progesterone receptor
qPCR: Quantitative polymerase chain reaction
