# Supplementary material for: Relationship between ER expression by IHC or mRNA with Ki67 response to aromatase inhibition: a POETIC study
Source: Breast Cancer Res. 2022 Sep 12;24:61. doi: 10.1186/s13058-022-01556-6 (PMC9466340; doi:10.1186/s13058-022-01556-6)
Supplement: Supplementary file 1 — Additional file 1: Supplementary Figures and Tables. [file 13058_2022_1556_MOESM1_ESM.pptx]

## Slide 1
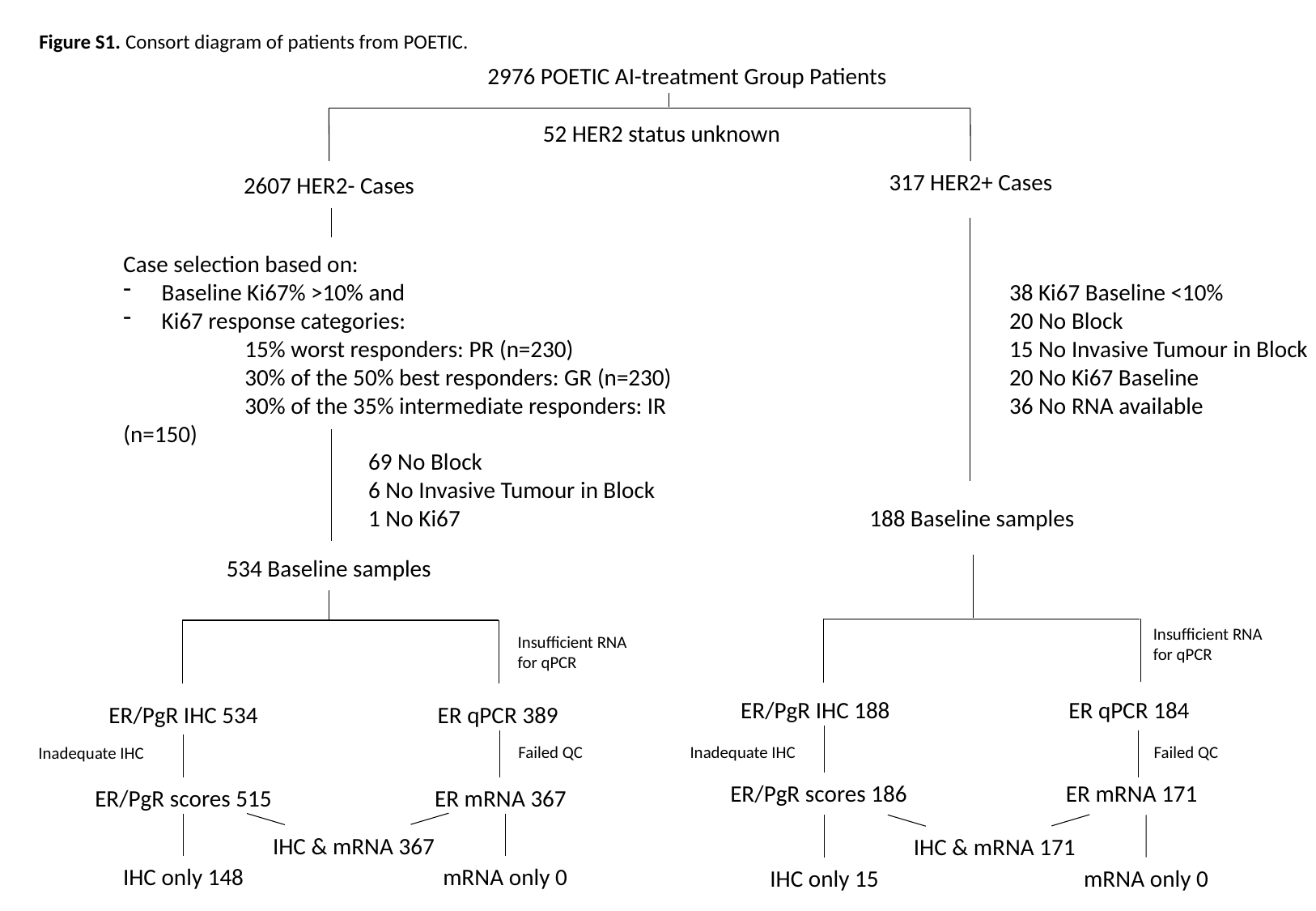

Figure S1. Consort diagram of patients from POETIC.
2976 POETIC AI-treatment Group Patients
52 HER2 status unknown
317 HER2+ Cases
2607 HER2- Cases
Case selection based on:
Baseline Ki67% >10% and
Ki67 response categories:
	15% worst responders: PR (n=230)
	30% of the 50% best responders: GR (n=230)
	30% of the 35% intermediate responders: IR (n=150)
38 Ki67 Baseline <10%
20 No Block
15 No Invasive Tumour in Block
20 No Ki67 Baseline
36 No RNA available
69 No Block
6 No Invasive Tumour in Block
1 No Ki67
188 Baseline samples
534 Baseline samples
Insufficient RNA for qPCR
Insufficient RNA for qPCR
ER/PgR IHC 188
ER qPCR 184
ER/PgR IHC 534
ER qPCR 389
Failed QC
Failed QC
Inadequate IHC
Inadequate IHC
ER/PgR scores 186
ER mRNA 171
ER/PgR scores 515
ER mRNA 367
IHC & mRNA 367
IHC & mRNA 171
IHC only 148
mRNA only 0
IHC only 15
mRNA only 0

## Slide 2
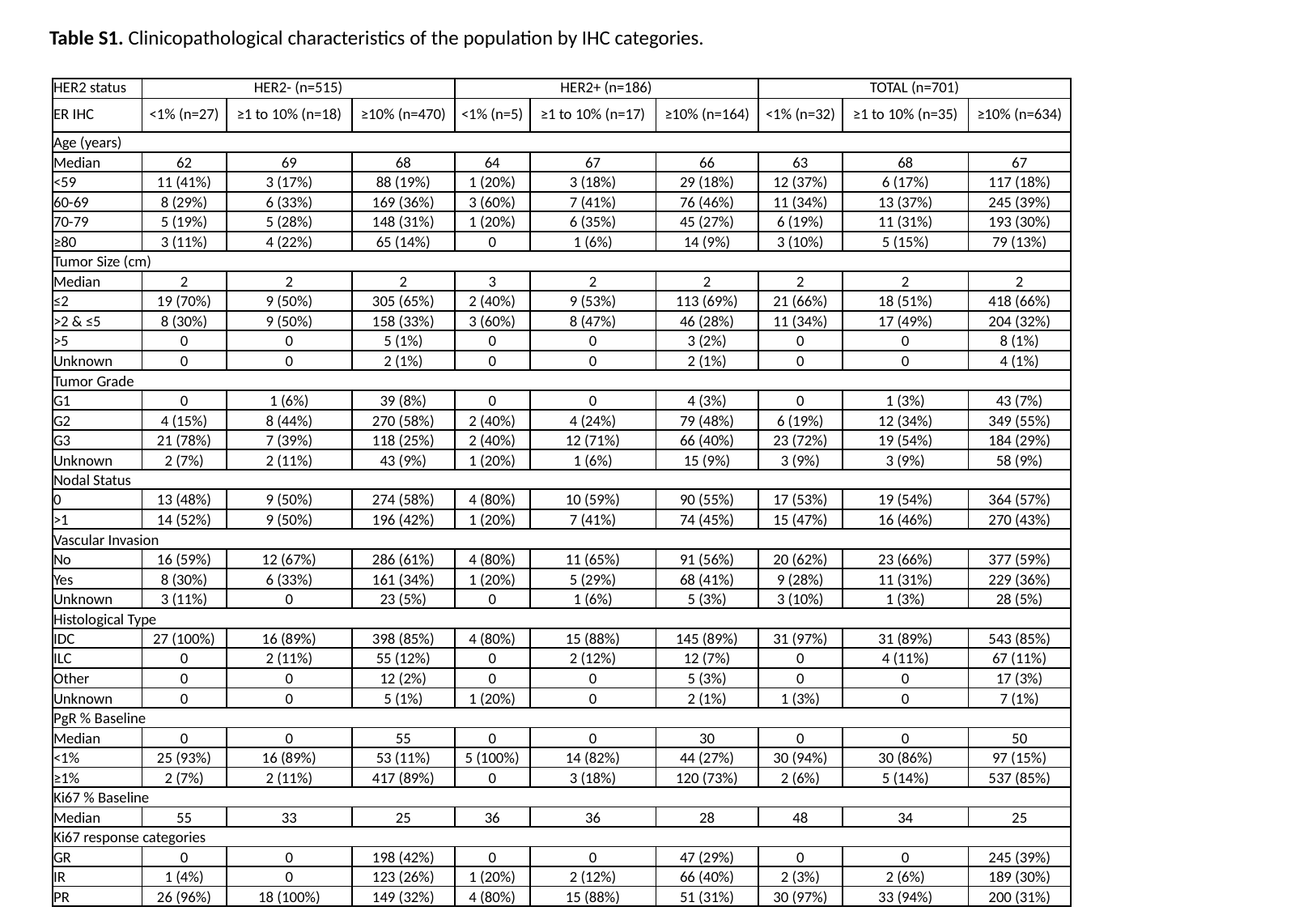

Table S1. Clinicopathological characteristics of the population by IHC categories.
| HER2 status | HER2- (n=515) | | | HER2+ (n=186) | | | TOTAL (n=701) | | |
| --- | --- | --- | --- | --- | --- | --- | --- | --- | --- |
| ER IHC | <1% (n=27) | ≥1 to 10% (n=18) | ≥10% (n=470) | <1% (n=5) | ≥1 to 10% (n=17) | ≥10% (n=164) | <1% (n=32) | ≥1 to 10% (n=35) | ≥10% (n=634) |
| Age (years) | | | | | | | | | |
| Median | 62 | 69 | 68 | 64 | 67 | 66 | 63 | 68 | 67 |
| <59 | 11 (41%) | 3 (17%) | 88 (19%) | 1 (20%) | 3 (18%) | 29 (18%) | 12 (37%) | 6 (17%) | 117 (18%) |
| 60-69 | 8 (29%) | 6 (33%) | 169 (36%) | 3 (60%) | 7 (41%) | 76 (46%) | 11 (34%) | 13 (37%) | 245 (39%) |
| 70-79 | 5 (19%) | 5 (28%) | 148 (31%) | 1 (20%) | 6 (35%) | 45 (27%) | 6 (19%) | 11 (31%) | 193 (30%) |
| ≥80 | 3 (11%) | 4 (22%) | 65 (14%) | 0 | 1 (6%) | 14 (9%) | 3 (10%) | 5 (15%) | 79 (13%) |
| Tumor Size (cm) | | | | | | | | | |
| Median | 2 | 2 | 2 | 3 | 2 | 2 | 2 | 2 | 2 |
| ≤2 | 19 (70%) | 9 (50%) | 305 (65%) | 2 (40%) | 9 (53%) | 113 (69%) | 21 (66%) | 18 (51%) | 418 (66%) |
| >2 & ≤5 | 8 (30%) | 9 (50%) | 158 (33%) | 3 (60%) | 8 (47%) | 46 (28%) | 11 (34%) | 17 (49%) | 204 (32%) |
| >5 | 0 | 0 | 5 (1%) | 0 | 0 | 3 (2%) | 0 | 0 | 8 (1%) |
| Unknown | 0 | 0 | 2 (1%) | 0 | 0 | 2 (1%) | 0 | 0 | 4 (1%) |
| Tumor Grade | | | | | | | | | |
| G1 | 0 | 1 (6%) | 39 (8%) | 0 | 0 | 4 (3%) | 0 | 1 (3%) | 43 (7%) |
| G2 | 4 (15%) | 8 (44%) | 270 (58%) | 2 (40%) | 4 (24%) | 79 (48%) | 6 (19%) | 12 (34%) | 349 (55%) |
| G3 | 21 (78%) | 7 (39%) | 118 (25%) | 2 (40%) | 12 (71%) | 66 (40%) | 23 (72%) | 19 (54%) | 184 (29%) |
| Unknown | 2 (7%) | 2 (11%) | 43 (9%) | 1 (20%) | 1 (6%) | 15 (9%) | 3 (9%) | 3 (9%) | 58 (9%) |
| Nodal Status | | | | | | | | | |
| 0 | 13 (48%) | 9 (50%) | 274 (58%) | 4 (80%) | 10 (59%) | 90 (55%) | 17 (53%) | 19 (54%) | 364 (57%) |
| >1 | 14 (52%) | 9 (50%) | 196 (42%) | 1 (20%) | 7 (41%) | 74 (45%) | 15 (47%) | 16 (46%) | 270 (43%) |
| Vascular Invasion | | | | | | | | | |
| No | 16 (59%) | 12 (67%) | 286 (61%) | 4 (80%) | 11 (65%) | 91 (56%) | 20 (62%) | 23 (66%) | 377 (59%) |
| Yes | 8 (30%) | 6 (33%) | 161 (34%) | 1 (20%) | 5 (29%) | 68 (41%) | 9 (28%) | 11 (31%) | 229 (36%) |
| Unknown | 3 (11%) | 0 | 23 (5%) | 0 | 1 (6%) | 5 (3%) | 3 (10%) | 1 (3%) | 28 (5%) |
| Histological Type | | | | | | | | | |
| IDC | 27 (100%) | 16 (89%) | 398 (85%) | 4 (80%) | 15 (88%) | 145 (89%) | 31 (97%) | 31 (89%) | 543 (85%) |
| ILC | 0 | 2 (11%) | 55 (12%) | 0 | 2 (12%) | 12 (7%) | 0 | 4 (11%) | 67 (11%) |
| Other | 0 | 0 | 12 (2%) | 0 | 0 | 5 (3%) | 0 | 0 | 17 (3%) |
| Unknown | 0 | 0 | 5 (1%) | 1 (20%) | 0 | 2 (1%) | 1 (3%) | 0 | 7 (1%) |
| PgR % Baseline | | | | | | | | | |
| Median | 0 | 0 | 55 | 0 | 0 | 30 | 0 | 0 | 50 |
| <1% | 25 (93%) | 16 (89%) | 53 (11%) | 5 (100%) | 14 (82%) | 44 (27%) | 30 (94%) | 30 (86%) | 97 (15%) |
| ≥1% | 2 (7%) | 2 (11%) | 417 (89%) | 0 | 3 (18%) | 120 (73%) | 2 (6%) | 5 (14%) | 537 (85%) |
| Ki67 % Baseline | | | | | | | | | |
| Median | 55 | 33 | 25 | 36 | 36 | 28 | 48 | 34 | 25 |
| Ki67 response categories | | | | | | | | | |
| GR | 0 | 0 | 198 (42%) | 0 | 0 | 47 (29%) | 0 | 0 | 245 (39%) |
| IR | 1 (4%) | 0 | 123 (26%) | 1 (20%) | 2 (12%) | 66 (40%) | 2 (3%) | 2 (6%) | 189 (30%) |
| PR | 26 (96%) | 18 (100%) | 149 (32%) | 4 (80%) | 15 (88%) | 51 (31%) | 30 (97%) | 33 (94%) | 200 (31%) |

## Slide 3
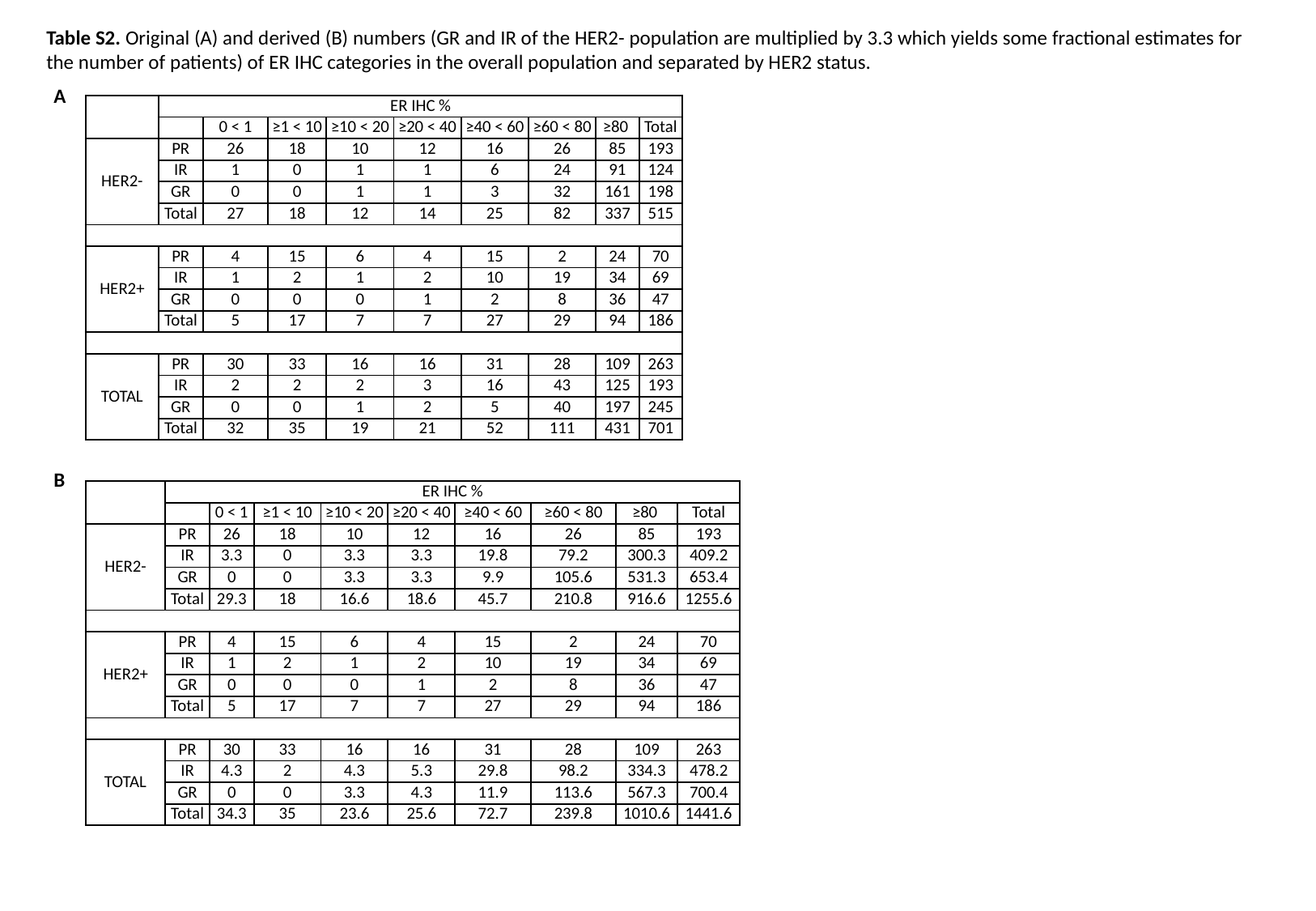

Table S2. Original (A) and derived (B) numbers (GR and IR of the HER2- population are multiplied by 3.3 which yields some fractional estimates for the number of patients) of ER IHC categories in the overall population and separated by HER2 status.
A
| | ER IHC % | | | | | | | | |
| --- | --- | --- | --- | --- | --- | --- | --- | --- | --- |
| | | 0 < 1 | ≥1 < 10 | ≥10 < 20 | ≥20 < 40 | ≥40 < 60 | ≥60 < 80 | ≥80 | Total |
| HER2- | PR | 26 | 18 | 10 | 12 | 16 | 26 | 85 | 193 |
| | IR | 1 | 0 | 1 | 1 | 6 | 24 | 91 | 124 |
| | GR | 0 | 0 | 1 | 1 | 3 | 32 | 161 | 198 |
| | Total | 27 | 18 | 12 | 14 | 25 | 82 | 337 | 515 |
| | | | | | | | | | |
| HER2+ | PR | 4 | 15 | 6 | 4 | 15 | 2 | 24 | 70 |
| | IR | 1 | 2 | 1 | 2 | 10 | 19 | 34 | 69 |
| | GR | 0 | 0 | 0 | 1 | 2 | 8 | 36 | 47 |
| | Total | 5 | 17 | 7 | 7 | 27 | 29 | 94 | 186 |
| | | | | | | | | | |
| TOTAL | PR | 30 | 33 | 16 | 16 | 31 | 28 | 109 | 263 |
| | IR | 2 | 2 | 2 | 3 | 16 | 43 | 125 | 193 |
| | GR | 0 | 0 | 1 | 2 | 5 | 40 | 197 | 245 |
| | Total | 32 | 35 | 19 | 21 | 52 | 111 | 431 | 701 |
B
| | ER IHC % | | | | | | | | |
| --- | --- | --- | --- | --- | --- | --- | --- | --- | --- |
| | | 0 < 1 | ≥1 < 10 | ≥10 < 20 | ≥20 < 40 | ≥40 < 60 | ≥60 < 80 | ≥80 | Total |
| HER2- | PR | 26 | 18 | 10 | 12 | 16 | 26 | 85 | 193 |
| | IR | 3.3 | 0 | 3.3 | 3.3 | 19.8 | 79.2 | 300.3 | 409.2 |
| | GR | 0 | 0 | 3.3 | 3.3 | 9.9 | 105.6 | 531.3 | 653.4 |
| | Total | 29.3 | 18 | 16.6 | 18.6 | 45.7 | 210.8 | 916.6 | 1255.6 |
| | | | | | | | | | |
| HER2+ | PR | 4 | 15 | 6 | 4 | 15 | 2 | 24 | 70 |
| | IR | 1 | 2 | 1 | 2 | 10 | 19 | 34 | 69 |
| | GR | 0 | 0 | 0 | 1 | 2 | 8 | 36 | 47 |
| | Total | 5 | 17 | 7 | 7 | 27 | 29 | 94 | 186 |
| | | | | | | | | | |
| TOTAL | PR | 30 | 33 | 16 | 16 | 31 | 28 | 109 | 263 |
| | IR | 4.3 | 2 | 4.3 | 5.3 | 29.8 | 98.2 | 334.3 | 478.2 |
| | GR | 0 | 0 | 3.3 | 4.3 | 11.9 | 113.6 | 567.3 | 700.4 |
| | Total | 34.3 | 35 | 23.6 | 25.6 | 72.7 | 239.8 | 1010.6 | 1441.6 |

## Slide 4
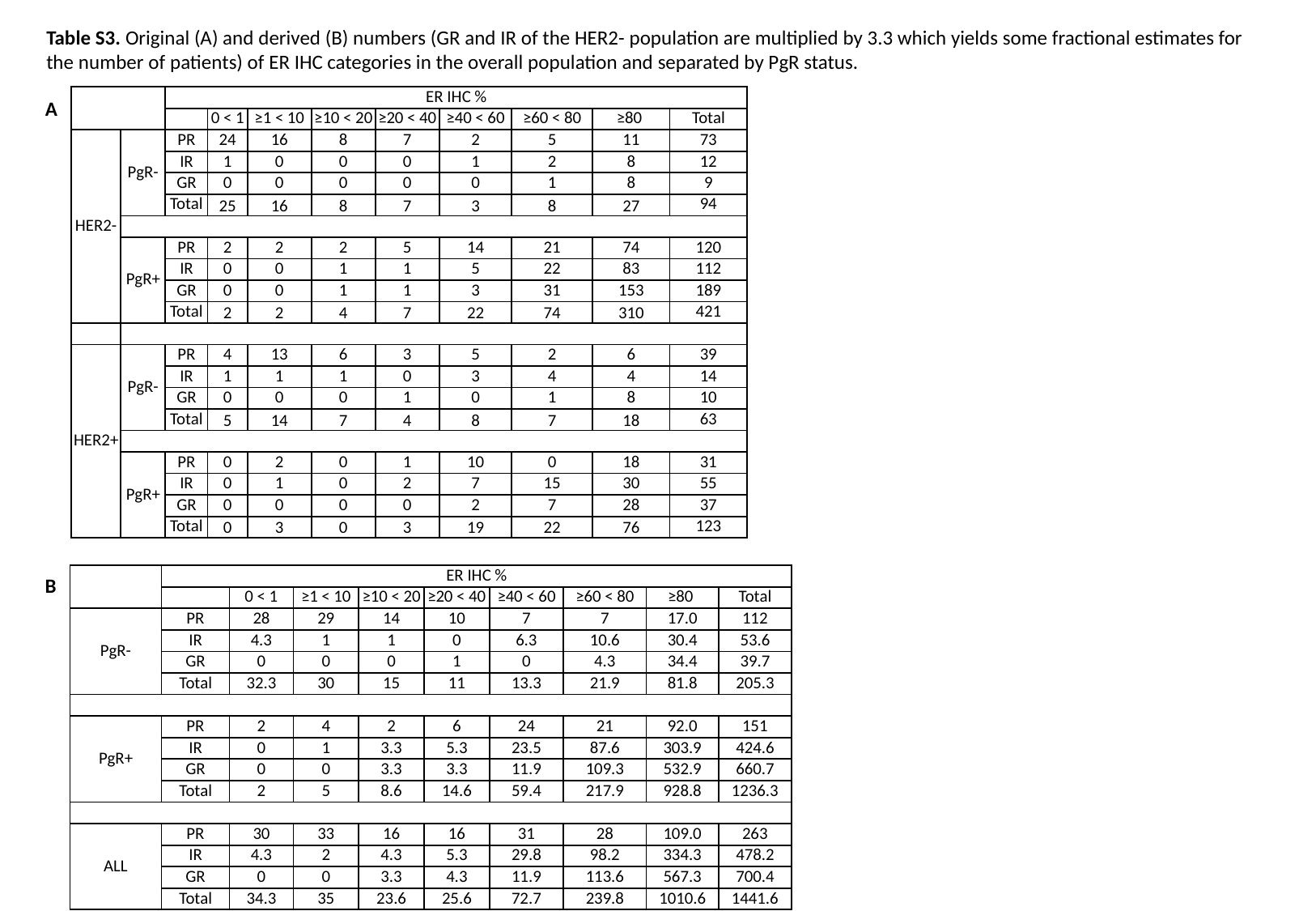

Table S3. Original (A) and derived (B) numbers (GR and IR of the HER2- population are multiplied by 3.3 which yields some fractional estimates for the number of patients) of ER IHC categories in the overall population and separated by PgR status.
| | | ER IHC % | | | | | | | | |
| --- | --- | --- | --- | --- | --- | --- | --- | --- | --- | --- |
| | | | 0 < 1 | ≥1 < 10 | ≥10 < 20 | ≥20 < 40 | ≥40 < 60 | ≥60 < 80 | ≥80 | Total |
| HER2- | PgR- | PR | 24 | 16 | 8 | 7 | 2 | 5 | 11 | 73 |
| | | IR | 1 | 0 | 0 | 0 | 1 | 2 | 8 | 12 |
| | | GR | 0 | 0 | 0 | 0 | 0 | 1 | 8 | 9 |
| | | Total | 25 | 16 | 8 | 7 | 3 | 8 | 27 | 94 |
| | | | | | | | | | | |
| | PgR+ | PR | 2 | 2 | 2 | 5 | 14 | 21 | 74 | 120 |
| | | IR | 0 | 0 | 1 | 1 | 5 | 22 | 83 | 112 |
| | | GR | 0 | 0 | 1 | 1 | 3 | 31 | 153 | 189 |
| | | Total | 2 | 2 | 4 | 7 | 22 | 74 | 310 | 421 |
| | | | | | | | | | | |
| HER2+ | PgR- | PR | 4 | 13 | 6 | 3 | 5 | 2 | 6 | 39 |
| | | IR | 1 | 1 | 1 | 0 | 3 | 4 | 4 | 14 |
| | | GR | 0 | 0 | 0 | 1 | 0 | 1 | 8 | 10 |
| | | Total | 5 | 14 | 7 | 4 | 8 | 7 | 18 | 63 |
| | | | | | | | | | | |
| | PgR+ | PR | 0 | 2 | 0 | 1 | 10 | 0 | 18 | 31 |
| | | IR | 0 | 1 | 0 | 2 | 7 | 15 | 30 | 55 |
| | | GR | 0 | 0 | 0 | 0 | 2 | 7 | 28 | 37 |
| | | Total | 0 | 3 | 0 | 3 | 19 | 22 | 76 | 123 |
A
| | ER IHC % | | | | | | | | |
| --- | --- | --- | --- | --- | --- | --- | --- | --- | --- |
| | | 0 < 1 | ≥1 < 10 | ≥10 < 20 | ≥20 < 40 | ≥40 < 60 | ≥60 < 80 | ≥80 | Total |
| PgR- | PR | 28 | 29 | 14 | 10 | 7 | 7 | 17.0 | 112 |
| | IR | 4.3 | 1 | 1 | 0 | 6.3 | 10.6 | 30.4 | 53.6 |
| | GR | 0 | 0 | 0 | 1 | 0 | 4.3 | 34.4 | 39.7 |
| | Total | 32.3 | 30 | 15 | 11 | 13.3 | 21.9 | 81.8 | 205.3 |
| | | | | | | | | | |
| PgR+ | PR | 2 | 4 | 2 | 6 | 24 | 21 | 92.0 | 151 |
| | IR | 0 | 1 | 3.3 | 5.3 | 23.5 | 87.6 | 303.9 | 424.6 |
| | GR | 0 | 0 | 3.3 | 3.3 | 11.9 | 109.3 | 532.9 | 660.7 |
| | Total | 2 | 5 | 8.6 | 14.6 | 59.4 | 217.9 | 928.8 | 1236.3 |
| | | | | | | | | | |
| ALL | PR | 30 | 33 | 16 | 16 | 31 | 28 | 109.0 | 263 |
| | IR | 4.3 | 2 | 4.3 | 5.3 | 29.8 | 98.2 | 334.3 | 478.2 |
| | GR | 0 | 0 | 3.3 | 4.3 | 11.9 | 113.6 | 567.3 | 700.4 |
| | Total | 34.3 | 35 | 23.6 | 25.6 | 72.7 | 239.8 | 1010.6 | 1441.6 |
B

## Slide 5
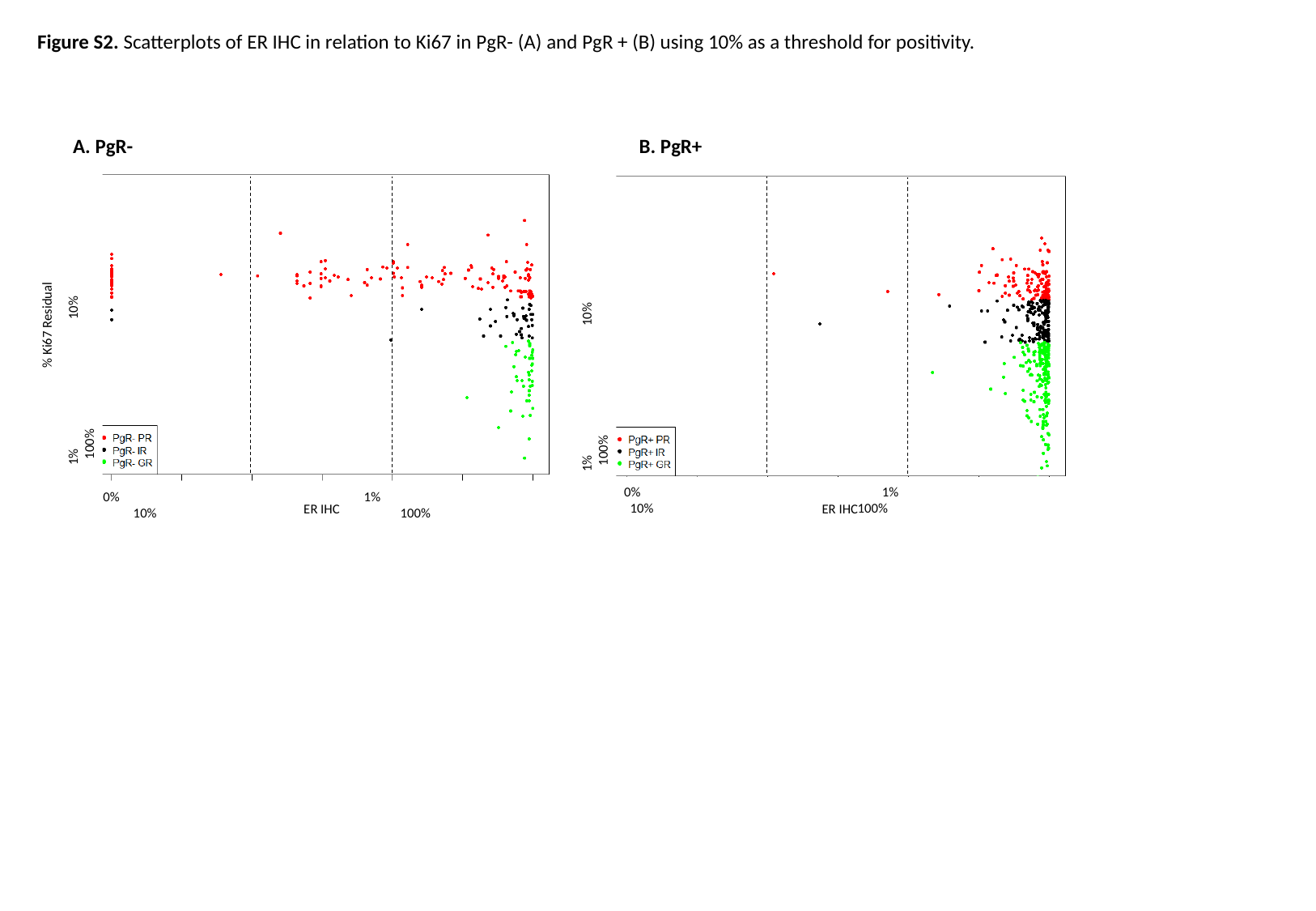

Figure S2. Scatterplots of ER IHC in relation to Ki67 in PgR- (A) and PgR + (B) using 10% as a threshold for positivity.
A. PgR-
B. PgR+
1%	 10%	 100%
% Ki67 Residual
1%	 10%	 100%
0%	 	 1%	 	 10%	 100%
0%	 	 1%	 	 10%	 	 100%
ER IHC
ER IHC

## Slide 6
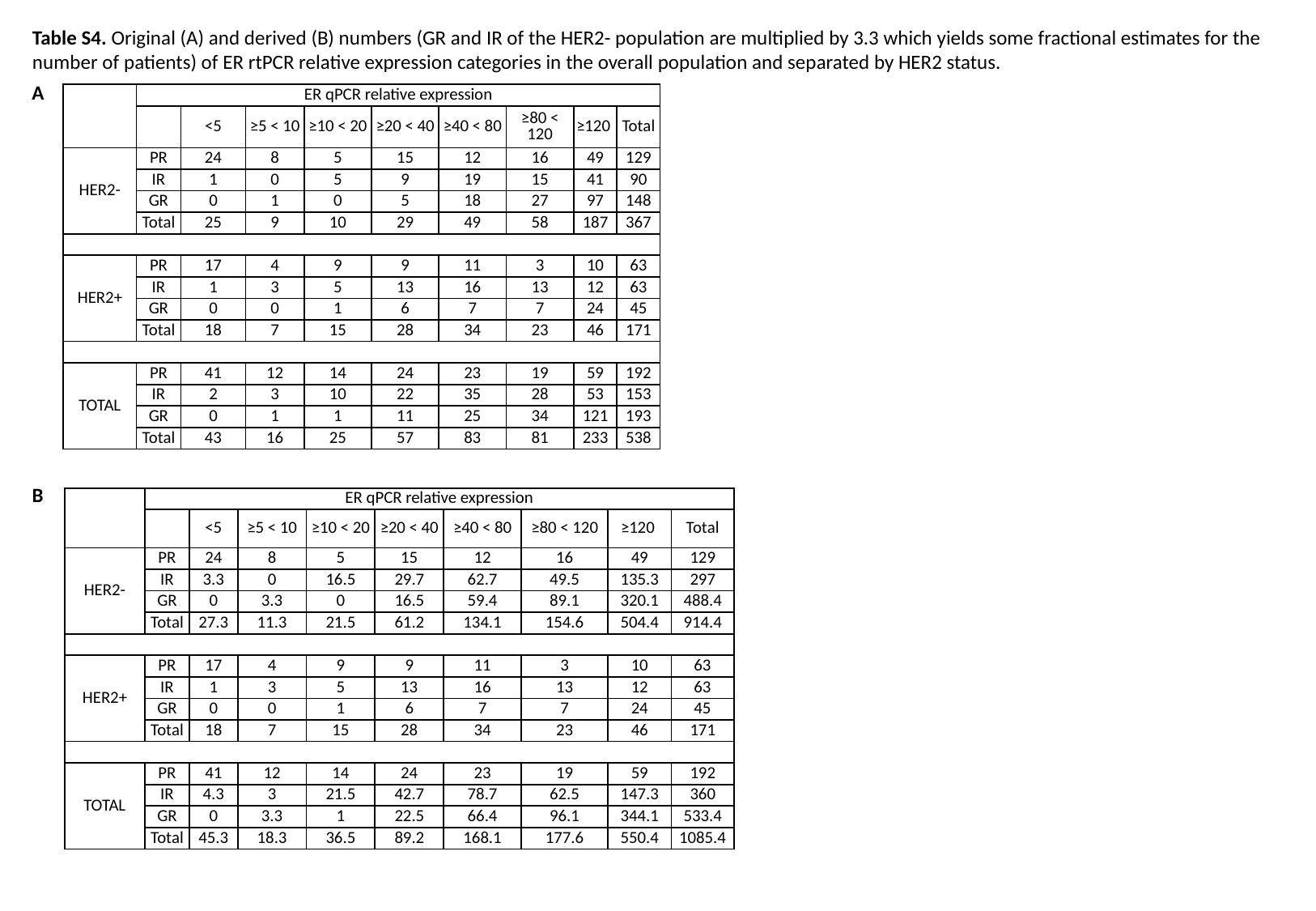

Table S4. Original (A) and derived (B) numbers (GR and IR of the HER2- population are multiplied by 3.3 which yields some fractional estimates for the number of patients) of ER rtPCR relative expression categories in the overall population and separated by HER2 status.
A
| | ER qPCR relative expression | | | | | | | | |
| --- | --- | --- | --- | --- | --- | --- | --- | --- | --- |
| | | <5 | ≥5 < 10 | ≥10 < 20 | ≥20 < 40 | ≥40 < 80 | ≥80 < 120 | ≥120 | Total |
| HER2- | PR | 24 | 8 | 5 | 15 | 12 | 16 | 49 | 129 |
| | IR | 1 | 0 | 5 | 9 | 19 | 15 | 41 | 90 |
| | GR | 0 | 1 | 0 | 5 | 18 | 27 | 97 | 148 |
| | Total | 25 | 9 | 10 | 29 | 49 | 58 | 187 | 367 |
| | | | | | | | | | |
| HER2+ | PR | 17 | 4 | 9 | 9 | 11 | 3 | 10 | 63 |
| | IR | 1 | 3 | 5 | 13 | 16 | 13 | 12 | 63 |
| | GR | 0 | 0 | 1 | 6 | 7 | 7 | 24 | 45 |
| | Total | 18 | 7 | 15 | 28 | 34 | 23 | 46 | 171 |
| | | | | | | | | | |
| TOTAL | PR | 41 | 12 | 14 | 24 | 23 | 19 | 59 | 192 |
| | IR | 2 | 3 | 10 | 22 | 35 | 28 | 53 | 153 |
| | GR | 0 | 1 | 1 | 11 | 25 | 34 | 121 | 193 |
| | Total | 43 | 16 | 25 | 57 | 83 | 81 | 233 | 538 |
B
| | ER qPCR relative expression | | | | | | | | |
| --- | --- | --- | --- | --- | --- | --- | --- | --- | --- |
| | | <5 | ≥5 < 10 | ≥10 < 20 | ≥20 < 40 | ≥40 < 80 | ≥80 < 120 | ≥120 | Total |
| HER2- | PR | 24 | 8 | 5 | 15 | 12 | 16 | 49 | 129 |
| | IR | 3.3 | 0 | 16.5 | 29.7 | 62.7 | 49.5 | 135.3 | 297 |
| | GR | 0 | 3.3 | 0 | 16.5 | 59.4 | 89.1 | 320.1 | 488.4 |
| | Total | 27.3 | 11.3 | 21.5 | 61.2 | 134.1 | 154.6 | 504.4 | 914.4 |
| | | | | | | | | | |
| HER2+ | PR | 17 | 4 | 9 | 9 | 11 | 3 | 10 | 63 |
| | IR | 1 | 3 | 5 | 13 | 16 | 13 | 12 | 63 |
| | GR | 0 | 0 | 1 | 6 | 7 | 7 | 24 | 45 |
| | Total | 18 | 7 | 15 | 28 | 34 | 23 | 46 | 171 |
| | | | | | | | | | |
| TOTAL | PR | 41 | 12 | 14 | 24 | 23 | 19 | 59 | 192 |
| | IR | 4.3 | 3 | 21.5 | 42.7 | 78.7 | 62.5 | 147.3 | 360 |
| | GR | 0 | 3.3 | 1 | 22.5 | 66.4 | 96.1 | 344.1 | 533.4 |
| | Total | 45.3 | 18.3 | 36.5 | 89.2 | 168.1 | 177.6 | 550.4 | 1085.4 |

## Slide 7
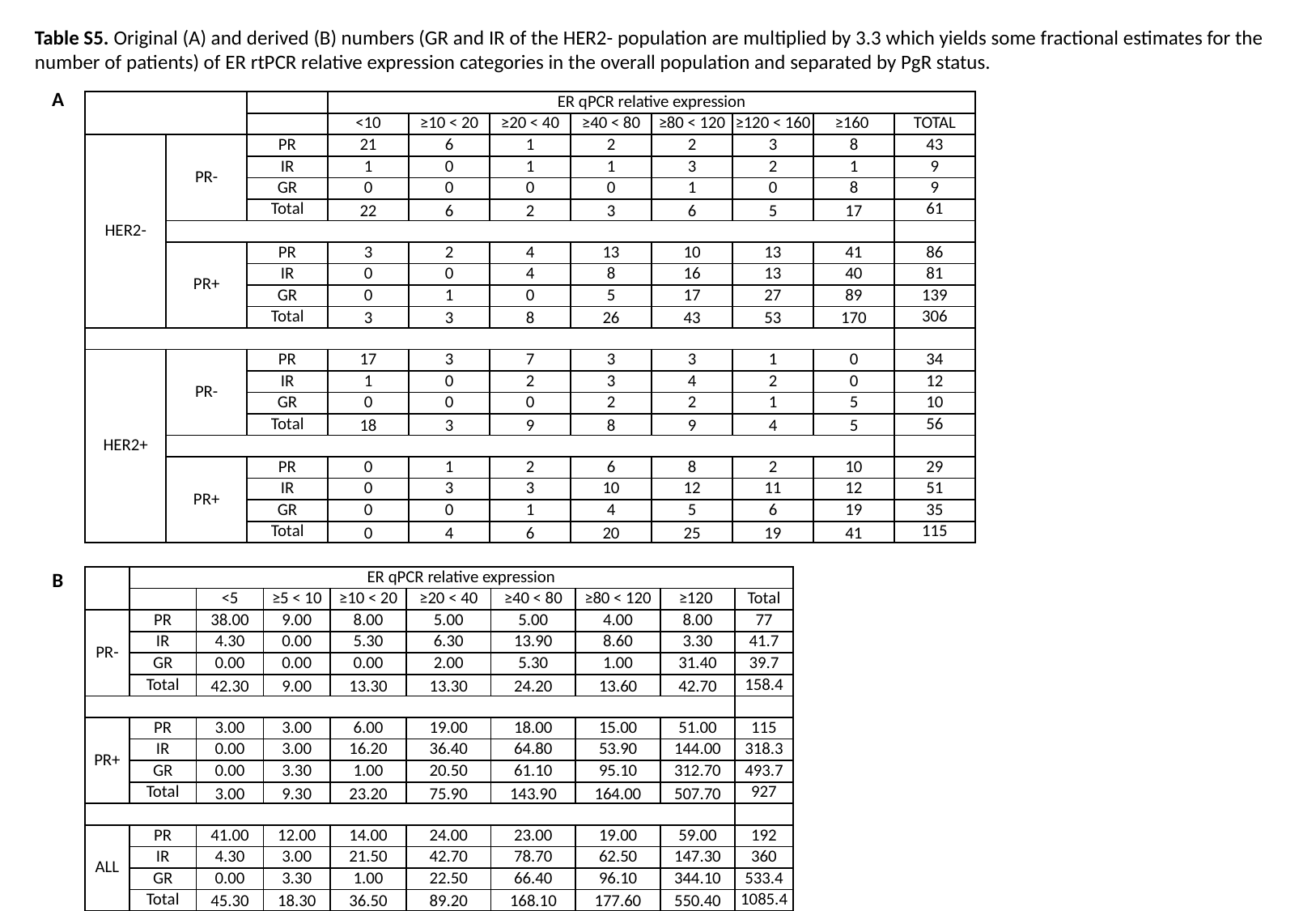

Table S5. Original (A) and derived (B) numbers (GR and IR of the HER2- population are multiplied by 3.3 which yields some fractional estimates for the number of patients) of ER rtPCR relative expression categories in the overall population and separated by PgR status.
A
| | | | ER qPCR relative expression | | | | | | | |
| --- | --- | --- | --- | --- | --- | --- | --- | --- | --- | --- |
| | | | <10 | ≥10 < 20 | ≥20 < 40 | ≥40 < 80 | ≥80 < 120 | ≥120 < 160 | ≥160 | TOTAL |
| HER2- | PR- | PR | 21 | 6 | 1 | 2 | 2 | 3 | 8 | 43 |
| | | IR | 1 | 0 | 1 | 1 | 3 | 2 | 1 | 9 |
| | | GR | 0 | 0 | 0 | 0 | 1 | 0 | 8 | 9 |
| | | Total | 22 | 6 | 2 | 3 | 6 | 5 | 17 | 61 |
| | | | | | | | | | | |
| | PR+ | PR | 3 | 2 | 4 | 13 | 10 | 13 | 41 | 86 |
| | | IR | 0 | 0 | 4 | 8 | 16 | 13 | 40 | 81 |
| | | GR | 0 | 1 | 0 | 5 | 17 | 27 | 89 | 139 |
| | | Total | 3 | 3 | 8 | 26 | 43 | 53 | 170 | 306 |
| | | | | | | | | | | |
| HER2+ | PR- | PR | 17 | 3 | 7 | 3 | 3 | 1 | 0 | 34 |
| | | IR | 1 | 0 | 2 | 3 | 4 | 2 | 0 | 12 |
| | | GR | 0 | 0 | 0 | 2 | 2 | 1 | 5 | 10 |
| | | Total | 18 | 3 | 9 | 8 | 9 | 4 | 5 | 56 |
| | | | | | | | | | | |
| | PR+ | PR | 0 | 1 | 2 | 6 | 8 | 2 | 10 | 29 |
| | | IR | 0 | 3 | 3 | 10 | 12 | 11 | 12 | 51 |
| | | GR | 0 | 0 | 1 | 4 | 5 | 6 | 19 | 35 |
| | | Total | 0 | 4 | 6 | 20 | 25 | 19 | 41 | 115 |
B
| | ER qPCR relative expression | | | | | | | | |
| --- | --- | --- | --- | --- | --- | --- | --- | --- | --- |
| | | <5 | ≥5 < 10 | ≥10 < 20 | ≥20 < 40 | ≥40 < 80 | ≥80 < 120 | ≥120 | Total |
| PR- | PR | 38.00 | 9.00 | 8.00 | 5.00 | 5.00 | 4.00 | 8.00 | 77 |
| | IR | 4.30 | 0.00 | 5.30 | 6.30 | 13.90 | 8.60 | 3.30 | 41.7 |
| | GR | 0.00 | 0.00 | 0.00 | 2.00 | 5.30 | 1.00 | 31.40 | 39.7 |
| | Total | 42.30 | 9.00 | 13.30 | 13.30 | 24.20 | 13.60 | 42.70 | 158.4 |
| | | | | | | | | | |
| PR+ | PR | 3.00 | 3.00 | 6.00 | 19.00 | 18.00 | 15.00 | 51.00 | 115 |
| | IR | 0.00 | 3.00 | 16.20 | 36.40 | 64.80 | 53.90 | 144.00 | 318.3 |
| | GR | 0.00 | 3.30 | 1.00 | 20.50 | 61.10 | 95.10 | 312.70 | 493.7 |
| | Total | 3.00 | 9.30 | 23.20 | 75.90 | 143.90 | 164.00 | 507.70 | 927 |
| | | | | | | | | | |
| ALL | PR | 41.00 | 12.00 | 14.00 | 24.00 | 23.00 | 19.00 | 59.00 | 192 |
| | IR | 4.30 | 3.00 | 21.50 | 42.70 | 78.70 | 62.50 | 147.30 | 360 |
| | GR | 0.00 | 3.30 | 1.00 | 22.50 | 66.40 | 96.10 | 344.10 | 533.4 |
| | Total | 45.30 | 18.30 | 36.50 | 89.20 | 168.10 | 177.60 | 550.40 | 1085.4 |

## Slide 8
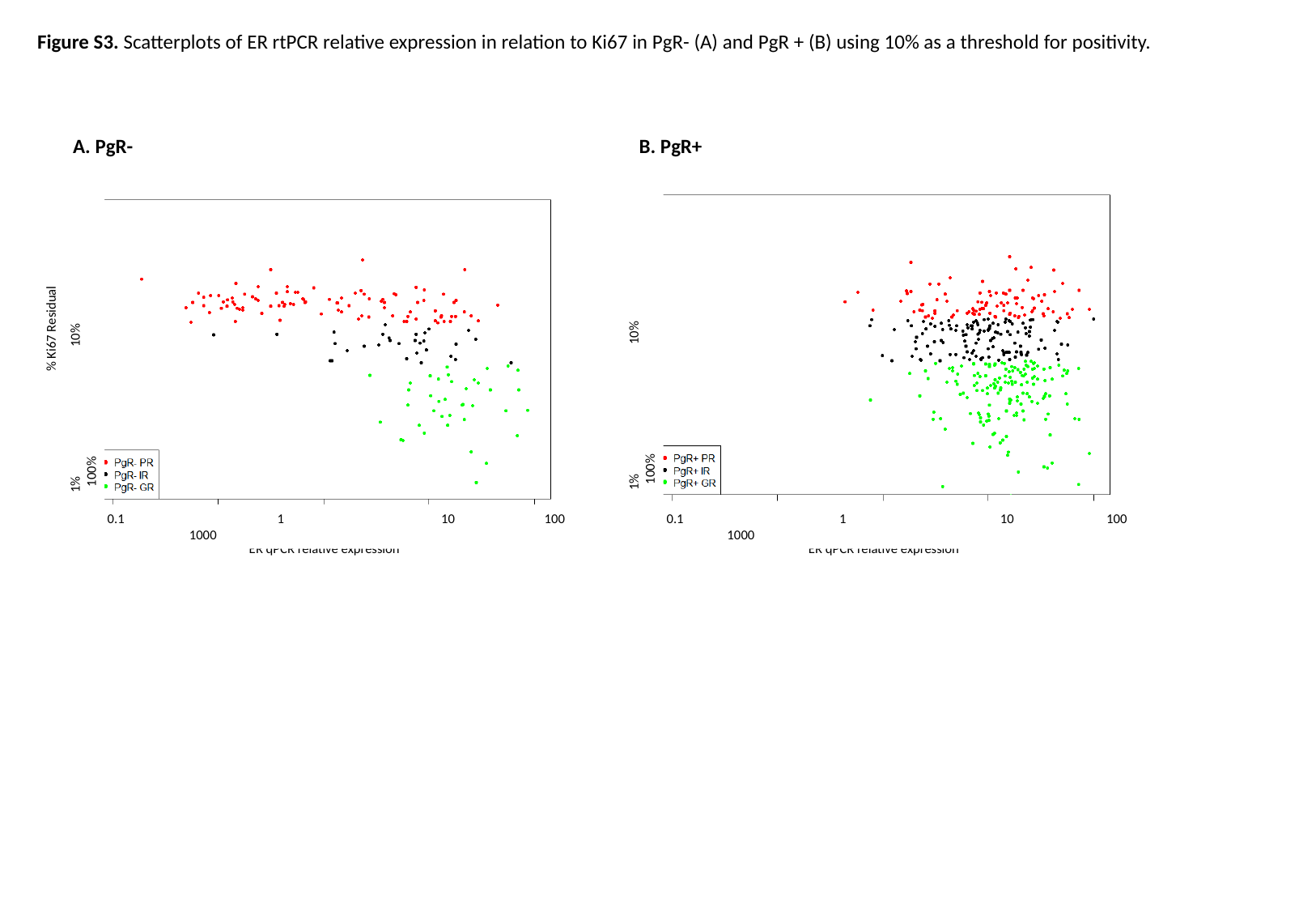

Figure S3. Scatterplots of ER rtPCR relative expression in relation to Ki67 in PgR- (A) and PgR + (B) using 10% as a threshold for positivity.
A. PgR-
B. PgR+
% Ki67 Residual
1%	 10%	 100%
1%	 10%	 100%
0.1	 1	 10	 100 1000
0.1	 1	 10	 100 1000
ER qPCR relative expression
ER qPCR relative expression
